# The Impact of Pediatric Dermatologic Conditions on Mental Health in Underserved Communities in the United States: Barriers and Strategies for Their Mitigation

**DOI:** 10.7759/cureus.113904

**Published:** 2026-08-03

**Authors:** Claudia Pedreira, Harvey N Mayrovitz

**Affiliations:** 1 Family Medicine, Nova Southeastern University Dr. Kiran C. Patel College of Osteopathic Medicine, Fort Lauderdale, USA; 2 Medical Education, Nova Southeastern University Dr. Kiran C. Patel College of Allopathic Medicine, Davie, USA

**Keywords:** access to care, atopic dermatitis, healthcare disparities, health equity, mental health, pediatric dermatology, pediatric skin disease, psychosocial burden, teledermatology, underserved populations

## Abstract

Pediatric dermatologic conditions are associated with significant psychosocial burden and disparities in access to care, particularly among underserved populations in the United States. Chronic skin diseases such as atopic dermatitis, acne, psoriasis, and alopecia areata may negatively affect emotional well-being, sleep, self-esteem, quality of life, and social functioning in affected children and caregivers. Structural inequities related to race, ethnicity, socioeconomic status, insurance coverage, transportation, and healthcare accessibility further contribute to differences in disease burden and treatment access. This narrative review examined the relationship between pediatric dermatologic conditions, mental health outcomes, healthcare disparities, and barriers to care in underserved pediatric populations. A structured search of PubMed, Web of Science, and PsycINFO identified peer-reviewed studies published between 2015 and 2025 involving pediatric patients aged 0-18 years. The reviewed literature demonstrated that underserved pediatric populations experience delayed dermatologic evaluation, reduced access to specialty care and advanced therapies, and increased emergency department utilization. Chronic pediatric skin disease was consistently associated with anxiety, depression, stigma, sleep disturbance, and impaired quality of life affecting both patients and caregivers. Teledermatology as an alternative care model may help improve specialty access and reduce barriers to care, although disparities in telehealth utilization remain. These findings highlight the complex relationship between pediatric dermatologic disease, psychosocial burden, and structural inequities affecting underserved children. Multidisciplinary, family-centered, and culturally responsive approaches may help improve both psychosocial outcomes and equitable access to pediatric dermatologic care.

## Introduction and background

Skin-related conditions are a common reason for healthcare utilization in pediatric populations, accounting for approximately 10% to 30% of primary care visits [1]. Over time, the number of physicians identifying as pediatric dermatologists in the United States has increased; however, this growth has remained insufficient to meet the clinical demand for specialized dermatologic care [1]. Pediatric dermatologic conditions encompass a wide range of diseases, with the most encountered including atopic dermatitis, acne vulgaris, contact dermatitis, viral warts, and impetigo [2,3]. The prevalence of these conditions varies by age group, with atopic dermatitis predominating in infancy and early childhood, while acne becomes more prevalent during adolescence [2,3]. Among these conditions, atopic dermatitis is one of the most common chronic inflammatory skin diseases in children and is associated with significant disease burden and increased healthcare costs [4]. Disease onset typically occurs early in life, with approximately 60% of affected children developing symptoms within the first year and nearly 80% by age six [4].

Beyond their physical manifestations, chronic pediatric skin disorders are associated with significant psychosocial impact, as visible disease can lead to stigmatization, which has been strongly correlated with depression, anxiety, and reduced quality of life in affected children [5]. This association is supported by large-scale evidence demonstrating that children and adolescents with atopic dermatitis have a significantly increased risk of multiple mental health disorders, including anxiety, depression, attention-deficit/hyperactivity disorder, and sleep disturbances [6]. These effects extend beyond the patient, as caregivers of children with atopic dermatitis report increased stress, while affected children also exhibit poorer sleep quality and higher rates of social-emotional developmental challenges [7].

Disparities in pediatric dermatology are well documented, as racial, ethnic, and socioeconomic factors contribute to differences in disease burden, access to care, and clinical outcomes among affected children [8]. These inequities reflect broader health disparities driven by complex interactions between social, economic, environmental, and healthcare system-level factors [9]. Within pediatric dermatology, such differences are particularly evident across racial, ethnic, and socioeconomic groups, with children from minority and low-income backgrounds experiencing higher prevalence and severity of atopic dermatitis, increased comorbidities, and reduced access to specialized dermatologic care [10,11]. These patterns are further reflected in care delivery, as children from minority and low-income backgrounds experience delayed access to treatment and higher rates of acute care utilization, including hospitalizations and urgent care visits [11]. Disparities also extend to gender-diverse populations, as transgender and gender-diverse youth experience higher rates of psychosocial stressors, including increased prevalence of mood and anxiety disorders, and acne may further compound this psychosocial burden [12]. Structural and systemic barriers further contribute to these differences, as limited availability of pediatric dermatologists, geographic maldistribution of specialists, insurance-related limitations, and factors such as language barriers, medical mistrust, and provider bias disproportionately affect children from minority and low-income backgrounds [10]. In addition, differences in treatment patterns and therapeutic access have been observed across racial and ethnic groups, with differences in the use of specific therapies such as topical calcineurin inhibitors and biologic agents like dupilumab among these populations [13].

Access to pediatric dermatologic care remains limited, with workforce shortages and geographic, sociocultural, and economic barriers contributing to significant challenges in obtaining timely specialty care [14]. In particular, while geographic maldistribution exists across the physician workforce, the limited and uneven distribution of pediatric dermatologists further restricts access to specialized care, especially for children living in rural and underserved communities [15]. These barriers are also evident at the systems level, as patients with public insurance, particularly those covered by Medicaid, face reduced access to dermatologic care, in part due to lower provider acceptance and higher rates of missed appointments, which have been associated with longer intervals between scheduling and appointment dates [16].

Teledermatology has expanded rapidly as a model of care, improving access to dermatologic services, reducing no-show rates, and demonstrating diagnostic accuracy comparable to in-person evaluations [17]. Despite these advantages, disparities in utilization persist, with underserved populations remaining less likely to use teledermatology [17]. Studies have also demonstrated high levels of patient and provider satisfaction with teledermatology, supporting its feasibility for the management of a wide range of pediatric dermatologic conditions [18]. Nonetheless, barriers to its use remain, including technological limitations and provider concerns regarding diagnostic confidence and quality of care compared to in-person evaluations, which may continue to impact equitable access [18,19].

Despite growing literature on pediatric dermatologic conditions, psychosocial burden, and healthcare disparities, these factors are often studied in isolation, with limited understanding of how they collectively impact patient outcomes, particularly in underserved populations. This review aims to examine how psychosocial burden and barriers to dermatologic care influence mental health outcomes in underserved children, while exploring teledermatology as a potential strategy to improve access and equity.

## Review

Methods

Search Strategy and Selection Criteria

A structured literature search was conducted using PubMed, Web of Science, and PsycINFO. This study was performed as a narrative review with a systematic search approach. Articles were limited to peer-reviewed publications in English published between 2015 and 2025 and focused on pediatric populations aged 0-18 years, consistent with standard definitions of children and adolescents. The search was conducted in September 2025. References published after completion of the literature search were cited selectively in the Introduction and Discussion to provide contemporary background and context but were not included in the systematic search, study selection, or narrative synthesis.

The search strategy combined controlled vocabulary and keywords using Boolean operators (AND, OR), including: "pediatric", "child", "adolescent", "eczema", "acne", "psoriasis", "vitiligo", "dermatology", "underserved", "Medicaid", "uninsured", "minority", "immigrant", "mental health", "psychosocial", "anxiety", "depression", and "quality of life".

Inclusion Criteria

Studies were included if they were peer-reviewed original research articles conducted in the United States, focused on pediatric populations (ages 0-18 years), examined dermatologic conditions, and addressed access to care, healthcare disparities, racial or socioeconomic inequities, or psychosocial and mental health outcomes.

Exclusion Criteria

Studies were excluded if they were conducted outside the United States, focused on non-dermatologic conditions, did not involve pediatric populations, or reported outcomes outside the scope of this review, including studies that did not assess access to care, healthcare disparities, or psychosocial and mental health outcomes, or that focused primarily on clinical management, treatment efficacy, or pathophysiology. Articles were also excluded if they were review articles, commentaries, editorials, clinical guidelines, case reports, conference abstracts, or other non-peer-reviewed publications.

The findings from the included studies were synthesized narratively according to recurring themes related to healthcare disparities, psychosocial burden, barriers to care, and teledermatology. Because of the heterogeneity of study designs and reported outcomes, no quantitative synthesis or meta-analysis was performed.

Results

Study Selection

**Figure 1:**
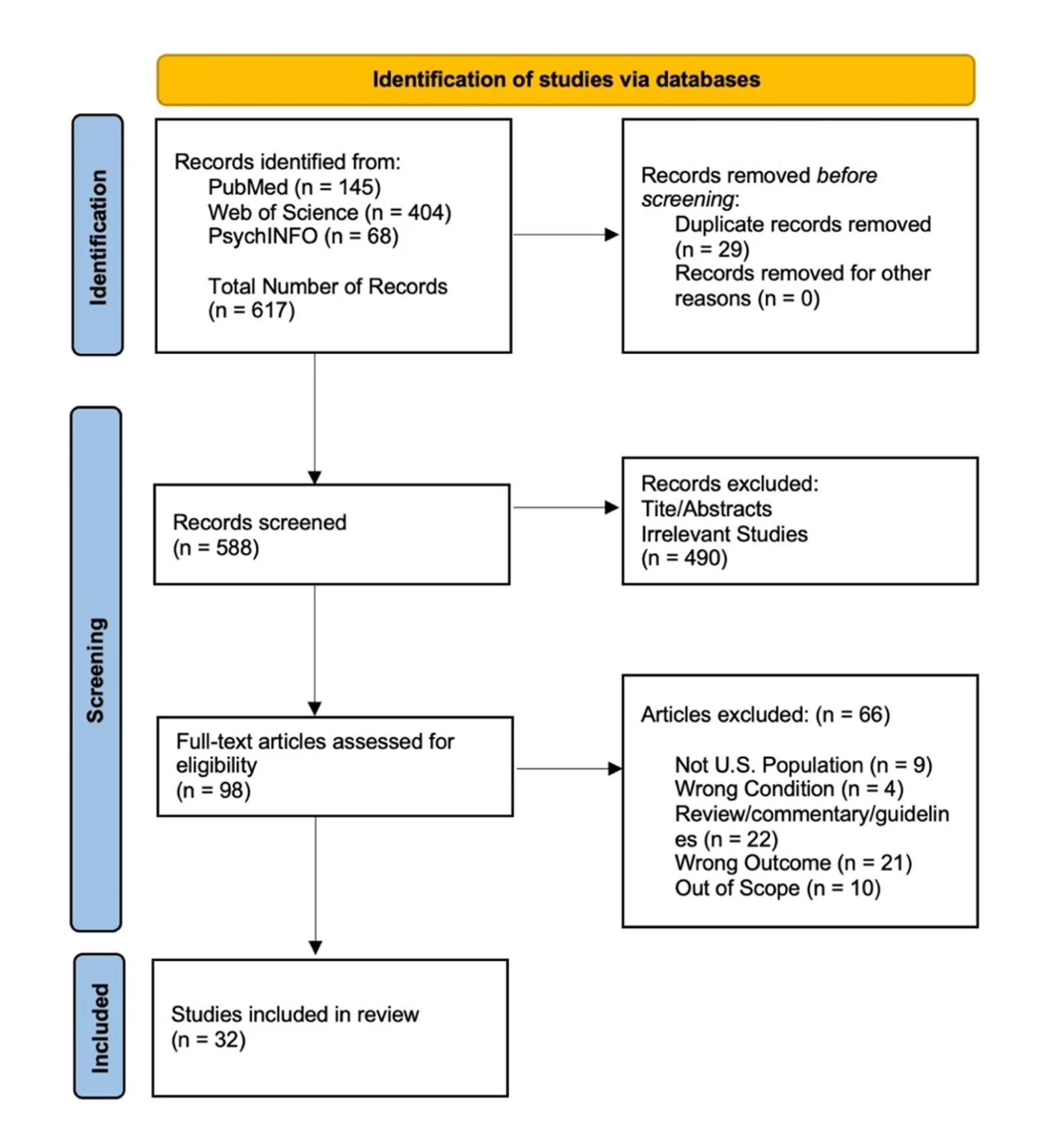
PRISMA flow diagram of study selection Preferred Reporting Items for Systematic Reviews and Meta-Analyses (PRISMA) flow diagram demonstrating the literature search and study selection process. Database searches identified 617 records, of which 588 remained after duplicate removal. Following title/abstract screening and full-text eligibility assessment, 32 studies met the predefined inclusion criteria and were included in the final narrative review.

The initial search yielded 617 articles. After removal of duplicates (n = 29) using the reference management software Zotero, 588 unique records were retained for title and abstract screening. Titles and abstracts were screened, followed by full-text review of eligible articles. A total of 98 full-text articles were assessed for eligibility. The study selection process is summarized using a Preferred Reporting Items for Systematic Reviews and Meta-Analyses (PRISMA) flow diagram (Figure 1). Ultimately, 32 studies were included in this review.

Study Characteristics

The included studies examined barriers to care and access disparities, racial and socioeconomic inequities, psychosocial and mental health impacts, and the unique needs of pediatric dermatology populations. Study designs included retrospective cohort studies, cross-sectional studies, qualitative studies, randomized controlled trials, and observational studies.

Racial, Ethnic, and Gender Inequities

Racial and ethnic disparities persist across pediatric dermatologic conditions, with minority children experiencing differences in disease burden and access to care. National data from 2006-2018 demonstrate disparities in healthcare utilization, with minority children showing lower or stagnant trends in visits to medical specialists and mental health professionals compared to their White and non-Hispanic peers [20]. Earlier data from 2001-2013 further demonstrate that non-Hispanic Black children have a higher prevalence of eczema compared to White and Hispanic children (17.1% vs 11.2% and 13.7%, respectively) and are less likely to have an ambulatory visit for eczema compared to White children [21]. Similarly, variations in treatment access have been observed, as Black and Hispanic children with atopic dermatitis had significantly lower odds of receiving dupilumab compared to non-Hispanic White children (odds ratio 0.43 and 0.46, respectively), even after adjusting for factors such as age, insurance status, and comorbidities [22]. In the United States, inequities in atopic dermatitis are evident early in life. Studies have shown that non-Hispanic Black and Hispanic infants experience delays in access to dermatologic care, including longer times to evaluation by a dermatologist and higher rates of emergency department visits compared to non-Hispanic White infants [23]. In addition, population-based data demonstrate that Black infants have a higher prevalence of atopic dermatitis by early childhood compared to White infants (37.0% vs 17.9%; odds ratio 2.6) [24].

Beyond eczema, racial and ethnic differences are also observed in other pediatric dermatologic conditions. Population-based data among children aged 5-17 years demonstrate that the prevalence of alopecia areata is higher among Hispanic, Black, and Asian/Pacific Islander children compared to non-Hispanic White children [25]. Similarly, dermatologic concerns are common among transgender and gender-diverse youth, including acne, androgenic alopecia, scarring from gender-affirming procedures, and eczema, with approximately 29.3% having at least one documented concern; however, referral rates to dermatology remain low, with only 9.3% of affected patients referred for evaluation [26].

Socioeconomic, Insurance-Based Inequities, and Family Structure

Socioeconomic status and insurance coverage are important determinants of pediatric dermatologic care, influencing access to specialty services and contributing to differences in treatment utilization and healthcare delivery [27,28]. Children from higher socioeconomic backgrounds were more frequently diagnosed with acne compared to age- and sex-matched peers, despite similar race and ethnicity distributions [29]. No association was found between socioeconomic status and age at diagnosis or type of treatment received [29].

Insurance status also contributes to unequal care, as children insured through Medicaid experienced longer wait times, fewer referrals to dermatologists, and less access to advanced therapies compared with those with private insurance [27]. In the same way, families with limited financial resources reported unfilled prescriptions and delayed follow-up visits due to cost concerns, as parents of children with atopic dermatitis had significantly higher odds of being unable to afford medications, follow-up care, or specialist visits, particularly when uninsured or low-income [30].

In addition to access, financial and insurance differences also shape what kind of care children receive. White and privately insured patients with molluscum contagiosum were more likely to see dermatologists and undergo procedures, while publicly insured and minority children were often managed by pediatricians who chose observation instead [28]. Family environment further adds to these disparities, as children from single-parent or blended families were more likely to develop atopic dermatitis, suggesting that stress and limited caregiver capacity may worsen symptoms and make regular care more difficult [31]. However, not all studies found a clear relationship between socioeconomic status and dermatologic outcomes. One study reported no significant association between socioeconomic factors and atopic dermatitis severity, demonstrating that the link between financial resources and disease outcomes may be more complex and influenced by other factors such as environment, access, or caregiver support [32].

Psychosocial Factors in Pediatric Dermatology

Children with chronic skin conditions face a significant psychosocial burden that goes beyond physical symptoms. In a national survey of over 228,000 children, those with atopic dermatitis (AD) showed higher rates of depression, attention deficit/hyperactivity disorder, behavioral issues, and frequent worry compared to their peers without AD, with these mental health problems increasing steadily with age [33]. Caregivers also report high emotional stress linked to the daily challenges of managing AD, including sleep loss that worsens with disease severity. More than half experience nightly sleep disruption and low energy, and many express worry, guilt, and social isolation because of their child's condition [34]. In a caregiver survey of pediatric AD patients, nearly half said their child had six or more days of poor mental health symptoms in the past month, with limited access to counseling due to provider shortages, transportation issues, and insurance barriers [35].

Psychosocial distress is also evident in other pediatric dermatologic conditions. In children and adolescents with psoriasis, both parents and patients described stigma, embarrassment, and anxiety related to visible lesions and treatment routines, with parents expressing concern about self-esteem, social withdrawal, and long-term psychosocial effects [36]. Among adolescents with acne, even mild cases showed quality-of-life impairment, and clinician-rated severity did not correlate with adolescent- or parent-reported impact [37]. Perceptions of acne severity and emotional effects also differed between adolescents, parents, and clinicians, highlighting gaps between clinical evaluation and patient experience [37].

Access and Barriers to Pediatric Dermatologic Care

Access to pediatric dermatologic care is influenced by multiple barriers, including transportation limitations, insurance-related challenges, and healthcare system constraints, as reported by primary care clinicians in underserved settings [38].

Medicaid-insured children face markedly reduced specialty access, requiring significantly longer travel distances and experiencing longer wait times between referral and completed appointment compared with privately insured peers [39]. Accuracy issues within Medicaid provider directories add to access challenges, with only 84.7% of listings correctly identifying board-certified pediatric dermatologists who accept Medicaid, and errors including both incorrect listings and omissions of eligible providers [40]. Treatment-specific limitations further contribute to inequities, as only half of pediatric respondents with vitiligo reported that narrowband ultraviolet B (UVB) phototherapy was offered, and those pursuing treatment frequently cited difficulty keeping appointments, missed school or work obligations, travel distance, and cost or insurance limitations [41].

Transportation barriers further contribute to inequitable care utilization, with approximately 279,000 US children with atopic dermatitis reporting delayed care due to lack of transportation annually, disproportionately affecting non-Hispanic Black and Hispanic children compared with non-Hispanic White children [42]. Longer travel distance is directly associated with reduced continuity of care, as children traveling more than 20 miles to a pediatric dermatology clinic are significantly more likely to be nonadherent to treatment recommendations and lost to follow-up compared with those living closer to care [43].

Caregivers describe several barriers to obtaining dermatology care for their children, most commonly difficulty securing timely appointments, with additional challenges including work conflicts, referral requirements, transportation limitations, and childcare needs [44]. Primary care clinicians similarly report that referral decisions are influenced by distance to specialty centers, insurance requirements, appointment availability, and additional factors such as language and financial strain [38]. In an academic pediatric dermatology clinic, median wait time from referral to appointment exceeds three months, and longer waits are associated with increased interim health care utilization and higher nonattendance rates [45].

Patterns of care utilization further reflect limited outpatient access, as pediatric atopic dermatitis visits in emergency settings occur disproportionately among Hispanic children and those with public insurance [46]. Among racially and ethnically minoritized families already connected to specialty care, parents emphasize that feeling "heard" by pediatric dermatology clinicians is central to trust, adherence, and continued engagement with care, whereas not feeling heard is associated with incomplete treatment and reduced follow-up [47].

Teledermatology and Alternative Care Models

Teledermatology has been associated with improved access to pediatric dermatology care and reduced wait times [48,49]. It has demonstrated the ability to provide accurate diagnosis through remote evaluation [50]. In a randomized clinical trial evaluating parent-submitted smartphone images across a range of pediatric dermatologic conditions, overall diagnostic concordance between photograph-based and in-person diagnoses was 83%, increasing to 89% when image quality was sufficient [50]. Diagnostic concordance was 92% for rashes, 100% for pigmented lesions and birthmarks, 64% for alopecia-related diagnoses, and 67% for nodular lesions [50]. Parents demonstrated strong willingness to use teledermatology services, with most respondents (80%) indicating willingness to pay for virtual evaluation, with a median willingness-to-pay of $20 [50].

Beyond direct-to-patient teledermatology, alternative care models such as electronic consultations (eConsults) have been used in pediatric dermatology care. eConsults are asynchronous, provider-to-provider communications conducted through the electronic medical record, allowing primary care clinicians to obtain specialist input without requiring an in-person visit. In a pediatric dermatology eConsult program, 95% of primary care physicians agreed that the service improved access to dermatology care, and 100% reported that participation enhanced their clinical knowledge [49].

Virtual group teledermatology models have also been used in pediatric dermatology care. In one study, virtual group visits were conducted via video platform and included patients with vitiligo and alopecia areata, with sessions consisting of group discussions and educational components led by a pediatric dermatologist without individualized medical management [51]. Before participation, 90% of children and 85% of parents reported knowing no one or few others with their condition. Additionally, 67% of children and 70% of parents reported having no one or few people who understood their condition prior to the visits, compared with 42% and 18% after participation, respectively. Following the sessions, 71% of children and 79% of parents reported willingness to attend future virtual group visits [51].

Teledermatology utilization did not differ significantly by race, ethnicity, socioeconomic status, or geographic location in a retrospective study of pediatric dermatology patients. However, patients whose primary language was not English were significantly less likely to engage in telehealth services [48].

The major challenges affecting pediatric dermatologic care identified throughout this review are summarized in Table 1.

**Table 1:** Major challenges affecting pediatric dermatologic care

Challenge	Key findings
Racial, ethnic, and gender inequities	Minority and gender-diverse pediatric populations experience differences in disease prevalence, healthcare utilization, access to dermatologic care, referral patterns, treatment utilization, and clinical outcomes.
Socioeconomic, insurance-based, and family-related inequities	Medicaid coverage, financial constraints, family structure, and insurance-related barriers contribute to delayed care, reduced access to specialists and advanced therapies, and difficulty obtaining medications and follow-up care.
Psychosocial burden	Chronic pediatric skin diseases are associated with anxiety, depression, impaired quality of life, sleep disturbances, stigma, and emotional stress among both children and caregivers. Caregivers also experience disrupted sleep, financial strain, and challenges managing ongoing treatment and healthcare appointments.
Access and barriers to pediatric dermatologic care	Workforce shortages, geographic maldistribution, transportation barriers, long wait times, referral requirements, language barriers, appointment availability, and healthcare system limitations continue to delay specialty evaluation and reduce continuity of care.
Teledermatology and alternative care models	Teledermatology and eConsults improve access to pediatric dermatologic care, reduce wait times, and demonstrate high diagnostic concordance; however, language differences and technology access continue to limit equitable utilization.

Discussion

Advancing Equity in Pediatric Dermatology

The findings from this narrative review reinforce the growing recognition that pediatric dermatologic conditions are associated with substantial psychosocial burden and that these effects may be amplified in underserved populations by persistent structural and healthcare-related inequities. Across the included studies, children from racial and ethnic minority groups, publicly insured families, rural communities, and socioeconomically disadvantaged backgrounds consistently experienced disparities in disease burden, access to specialty care, treatment utilization, and mental health outcomes. Collectively, these findings suggest that pediatric dermatologic disease should not be viewed solely as a cutaneous condition, but rather as a multifactorial health issue shaped by social, psychological, and systemic influences.

Racial, ethnic, and socioeconomic disparities remain a persistent feature of pediatric dermatologic care and appear to influence both disease burden and healthcare utilization among affected children. Minority children experienced higher prevalence of chronic inflammatory skin conditions, delays in dermatologic evaluation, increased emergency department utilization, and reduced access to advanced therapies compared with White and privately insured peers [20-24]. Similar inequities were also observed among publicly insured and gender-diverse pediatric populations, suggesting that disparities in pediatric dermatology extend beyond differences in disease prevalence alone and may influence how children access and engage with healthcare systems [26-30]. These disparities may also extend to clinical recognition and diagnosis in children with skin of color. Dermatologic conditions may present differently in more heavily pigmented skin, with erythema often being less apparent and inflammatory dermatoses demonstrating more prominent pigmentary changes, scaling, lichenification, or distinct morphologic patterns in some populations. Increased pigmentation may also obscure characteristic clinical and dermoscopic findings, potentially complicating recognition among clinicians with limited exposure to dermatologic disease across diverse skin tones [52]. Garcia-Creighton et al. further highlighted the challenges racially and ethnically minoritized families encounter when seeking pediatric dermatologic care, including barriers related to healthcare navigation, appointment availability, communication, and trust in clinical interactions. The authors additionally noted that limited access to specialty services may contribute to delayed treatment escalation and worsening disease burden among underserved children [53]. These findings suggest that inequities in pediatric dermatology remain closely tied to broader structural and socioeconomic disparities affecting vulnerable populations.

The Hidden Burden of Chronic Skin Disease

Psychosocial burden appears to be a major component of pediatric dermatologic disease and frequently extends beyond the visible manifestations of skin conditions. Across multiple studies, chronic pediatric skin disease was associated with impaired quality of life, emotional distress, stigma, sleep disruption, and social isolation affecting both patients and caregivers [33-37]. Several studies additionally demonstrated discrepancies between clinician-assessed disease severity and patient- or caregiver-perceived psychosocial impact, suggesting that emotional burden may not be fully captured during routine clinical evaluation [36,37]. This finding is particularly important given that visible dermatologic disease develops during periods of social and emotional development in childhood and adolescence, when self-image, peer relationships, and social integration may be especially vulnerable to disruption. Recent psychodermatology literature further supports the complex relationship between chronic skin disease and mental health, emphasizing impairments in peer relationships, school functioning, emotional regulation, and long-term quality of life [54]. Similarly, caregiver-related stressors, including parental anxiety, financial strain, and family stress, may contribute to inconsistent treatment adherence and worsening disease management in pediatric atopic dermatitis [55]. Family burden may additionally extend into sleep impairment, strained interpersonal relationships, reduced work productivity, and social isolation among caregivers and family members [56]. These findings support the importance of more integrated and family-centered approaches that address both the physical and psychosocial dimensions of pediatric dermatologic disease.

Overcoming Barriers to Specialty Care

Barriers to pediatric dermatologic care remain closely linked to broader socioeconomic and healthcare system inequities. Families frequently reported difficulty obtaining timely dermatology appointments, while transportation limitations, insurance-related restrictions, financial strain, and competing caregiving responsibilities further complicated access to specialty care. Medicaid-insured children additionally experienced longer travel distances, prolonged wait times, and reduced access to advanced therapies compared with privately insured peers [39-41]. These findings suggest that healthcare access is influenced not only by disease burden, but also by structural limitations that may disproportionately affect underserved populations. Oluwole et al. further emphasized that poverty, housing insecurity, food insecurity, transportation barriers, and adverse childhood experiences may negatively affect healthcare access, treatment adherence, and disease severity among children with chronic dermatologic conditions [57]. The authors additionally noted that children are particularly vulnerable to these inequities because they often depend on caregivers to navigate healthcare systems and maintain continuity of care [57]. Workforce shortages may further contribute to these barriers. Sinha et al. demonstrated substantial urban-rural maldistribution within the pediatric dermatology workforce, with most specialists concentrated in urban centers and several states lacking any board-certified pediatric dermatologist entirely [58]. Together, these findings highlight how structural, geographic, and socioeconomic inequities may collectively limit access to pediatric dermatologic care for vulnerable populations.

The Future of Teledermatology

Teledermatology has emerged as a promising strategy to improve access to pediatric dermatologic care, particularly for underserved and geographically isolated populations. Across several studies, teledermatology was associated with reduced wait times, improved access to specialty evaluation, and high diagnostic concordance for common pediatric dermatologic conditions [48-50]. eConsult systems and parent-submitted image models also demonstrated strong caregiver and primary care clinician acceptance, suggesting that remote care models may help reduce delays related to travel, referral requirements, and limited specialist availability [49,50]. Importantly, the benefits of teledermatology appeared to extend beyond logistical convenience alone. Virtual group visit models demonstrated improved feelings of social support and disease understanding among both children and caregivers, highlighting the potential for teledermatology to address psychosocial isolation associated with chronic pediatric skin disease [51]. Uscher-Pines et al. similarly demonstrated that practices using teledermatology experienced substantially greater increases in dermatology utilization among Medicaid populations compared with practices that did not use teledermatology [59]. Teledermatology additionally served higher proportions of pediatric, nonwhite, and Medicaid-enrolled patients, populations that have historically experienced greater barriers to dermatologic care [59]. However, disparities in telehealth engagement may still persist. Lower utilization among families whose primary language was not English highlights the continued influence of language barriers, digital accessibility, and healthcare navigation challenges even within remote care models [48]. These findings suggest that teledermatology may serve as an important adjunct to traditional pediatric dermatology care while also emphasizing the need for equitable implementation strategies that address persistent barriers to healthcare access.

Clinical Implications and Future Directions

The findings summarized in this review have important implications for the delivery of pediatric dermatologic care, particularly among underserved populations. Chronic pediatric skin disease appears to affect not only physical health, but also emotional well-being, family functioning, treatment adherence, and healthcare utilization. Greater attention toward psychosocial screening, culturally responsive care, and multidisciplinary support may help improve both clinical outcomes and quality of life among affected children and families. Emerging artificial intelligence (AI)-assisted technologies may further complement these approaches by supporting image analysis and teledermatology workflows. However, pediatric-specific validation remains necessary before widespread clinical implementation, as many AI models have been developed using predominantly adult datasets and require evaluation across different pediatric age groups and diverse patient populations [60].

Limitations

This narrative review has several limitations. First, the narrative design and heterogeneity of included studies prevented quantitative synthesis, and a formal risk-of-bias assessment of individual studies was not performed. Additionally, many studies were cross-sectional, survey-based, or conducted at single academic centers, potentially limiting generalizability across broader pediatric populations. Variability in study design, outcome measures, and definitions of psychosocial burden also complicated direct comparison between studies. Several studies focused primarily on specific conditions, including atopic dermatitis, which may limit applicability across the full spectrum of pediatric dermatologic disease.

Despite these limitations, consistent patterns emerged across the literature supporting the importance of psychosocial, structural, and healthcare-related factors in pediatric dermatologic outcomes. Future research should prioritize larger longitudinal studies examining how disparities in access, treatment adherence, and psychosocial burden evolve across childhood and adolescence. Additional investigation into integrated psychodermatology models, culturally responsive interventions, standardized psychosocial screening tools, and equitable teledermatology implementation strategies may help improve comprehensive care for children with chronic dermatologic conditions.

## Conclusions

Pediatric dermatologic conditions are associated with substantial psychosocial burden and persistent inequities in access to care that may disproportionately affect underserved populations. Although chronic pediatric skin disease is often approached primarily through a dermatologic framework, the literature demonstrates that these conditions may also significantly influence emotional well-being, family functioning, treatment adherence, and quality of life. Structural barriers related to socioeconomic status, insurance coverage, transportation, workforce distribution, and healthcare accessibility continue to contribute to disparities in pediatric dermatologic care.

Emerging evidence suggests that teledermatology and alternative models of care may help improve specialty access and reduce some of these barriers, particularly among geographically isolated and publicly insured populations. However, disparities in healthcare engagement and psychosocial support remain prevalent. Collectively, these findings underscore the importance of multidisciplinary, family-centered, and culturally responsive approaches to pediatric dermatologic care that address both the physical and psychosocial dimensions of disease. Future research should prioritize longitudinal and intervention-based studies aimed at improving equitable access to care, strengthening psychosocial support systems, and evaluating the long-term effectiveness of integrated and teledermatology-based care models.
